# A Comparative Study of Efficacy and Functionality of Ten Commercially Available Wrist-Hand Orthoses in Healthy Females: Wrist Range of Motion and Grip Strength Analysis

**DOI:** 10.3389/fresc.2021.687554

**Published:** 2021-06-15

**Authors:** Alejandra Aranceta-Garza, Karyn Ross, Miranda Buhler, Eugene Rameckers

**Affiliations:** Department of Biomedical Engineering, University of Strathclyde, Glasgow, United Kingdom; University of Otago, New Zealand; University of Hasselt, Belgium

**Keywords:** effectiveness, range of motion, grip strength, pre-fabricated wrist hand orthosis, wrist splint

## Abstract

**Objective:**

Wrist-hand orthoses (WHOs) are prescribed for a range of musculoskeletal/neurological conditions to optimise wrist/hand position at rest and enhance performance by controlling its range of motion (ROM), improving alignment, reducing pain, and optimising grip strength. The objective of this research was to study the efficacy and functionality of ten commercially available WHOs on wrist ROM and grip strength.

**Design:**

Randomised comparative functional study of the wrist/hand with and without WHOs.

**Participants:**

Ten right-handed female participants presenting with no underlying condition nor pain affecting the wrist/hand which could influence motion or grip strength. Each participant randomly tested ten WHOs; one per week, for 10 weeks.

**Main outcome measures:**

The primary outcome was to ascertain the impact of WHOs on wrist resting position and flexion, extension, radial, and ulnar deviation. A secondary outcome was the impact of the WHOs on maximum grip strength and associated wrist position when this was attained.

**Results:**

From the 2,400 tests performed it was clear that no WHO performed effectively or consistently across participants. The optimally performing WHO for flexion control was #3 restricting 86.7%, #4 restricting 76.7% of extension, #9 restricting 83.5% of radial deviation, and #4 maximally restricting ulnar deviation. A grip strength reduction was observed with all WHOs, and ranged from 1.7% (#6) to 34.2% (#4).

**Conclusion:**

WHOs did not limit movement sufficiently to successfully manage any condition requiring motion restriction associated with pain relief. The array of motion control recorded might be a contributing factor for the current conflicting evidence of efficacy for WHOs. Any detrimental impact on grip strength will influence the types of activities undertaken by the wearer. The design aspects impacting wrist motion and grip strength are multifactorial, including: WHO geometry; the presence of a volar bar; material of construction; strap design; and quality of fit. This study raises questions regarding the efficacy of current designs of prefabricated WHOs which have remained unchanged for several decades but continue to be used globally without a robust evidence-base to inform clinical practise and the prescription of these devices. These findings justify the need to re-design WHOs with the goal of meeting users’ needs.

## Introduction

Optimal hand function depends on many interrelated factors, including optimal skeletal integrity, joint alignment, muscle, and neurological function. All of these may be affected by rheumatological musculoskeletal and neurological conditions or injuries. Rheumatological conditions affecting the hand and the wrist are associated with over 150 diseases and syndromes that frequently progress with time and are often concurrent with pain (1). Around 1% of the UK population (over 400,000 people) has rheumatoid arthritis (RA) with clinical features of synovitis, including pain, swelling, heat, and stiffness in the affected joints impacting negatively on quality of life (2). The wrist and hand are commonly affected in the early stages of RA with 85% of people reporting hand involvement during the first year of the disease (1). Functional wrist-hand orthoses (WHOs) or “working splints” as they are often referred to, are used in the conservative management of a wide variety of conditions managed within the acute and primary care sectors but are also used prophylactically in the workplace and during sporting activities. They are commonly prescribed and fitted by healthcare providers, but are also available on the high street, and through online retailers. As such, it is difficult to ascertain the annual cost associated with procurement/provision of WHOs. In the US, the annual conservative estimate of Medicare reimbursement within the 65+ population for the provision of these types of WHOs is $22 million, so when extrapolated to the UK population, it represents an annual cost of £3.9 million.

In general, WHOs are often prescribed to maintain the wrist in a functional position of ~10 to 15° of extension and prevent movement into flexion during activity (3). Ideally, they should only provide control of the wrist while enabling free movement of the fingers and the thumb to facilitate optimal hand function during activities of daily living (ADLs). In those with longstanding RA which affects the hands, the wrist often adopts a position of flexion, resulting in altered biomechanics and further compromising the efficiency of the flexor muscles (4). It is widely considered that a WHO that controls and restricts wrist flexion will reduce pain associated with synovitis and the improved position of wrist extension will increase the mechanical advantage of the finger flexor muscles, thereby improving hand function (5). Additionally, with RA, the wrist may assume a position of radial deviation with associated ulnar drift of the fingers which further negatively impacts hand function and grip strength. Therefore, ideally the WHO should also control the radial deviation of the wrist for this population but is rarely integrated into the design of prefabricated WHOs.

Maximum power grip strength is achieved with the wrist in a position of extension of between 15 and 30° (6, 7) and although not conclusive in the literature, strong evidence suggests that a position of up to 15° of ulnar deviation is also required to achieve this (6, 8). Of interest, if the wrist is positioned at a greater amount of extension (>15°) with a WHO, this may have a negative impact on carrying out many ADLs. While a combination of extension and ulnar deviation optimises the ability to exert a maximum power grip strength, it has also been demonstrated that a combination of flexion and ulnar deviation in addition to being detrimental to power grip, is also associated with increasing levels of pain (9).

As prefabricated WHOs aim to target a range of conditions, the quantification of the efficacy and performance is challenging. Previous studies have investigated the efficacy of functional WHOs in the RA population, reporting impact on: (i) pain (10–20); (ii) grip or pinch strength (11–13, 19–23); and (iii) dexterity and performance during everyday functional tasks (14, 16, 21–24). However, there is considerable variability across the studies, with many contradictory findings which may be attributable to several factors, such as:

i) inconsistencies and/or incomplete reporting of the methodology (25);
ii) inconsistent and/or incomplete reporting of participant characteristics;
iii) inconsistent and/or incomplete reporting of the wrist hand orthosis/es tested;
iv) the contour and fit of the WHO in the palmar region impeding grip patterns and grip strength; and/or
v) suboptimal wrist movement control in the sagittal place, which could be related to design issues (e.g., limitations associated with the materials from which the WHOs are constructed; WHOs’ design and geometry; overall fit and securing of the WHO to the upper limb).

In the absence of robust research protocols which investigate the true motion control of the wrist provided by functional WHOs and examine the impact of the design characteristics on functionality, it is challenging for healthcare professionals to advocate the use of a specific device.

The main aim of this study was to investigate, test and compare the efficacy and functionality of ten commercially available pre-fabricated WHOs, with a specific emphasis on motion control of the wrist and grip strength.

## Materials And Methods

The research team developed a robust, comprehensive, and repeatable testing protocol to assess the parameters consistently across ten commercially available prefabricated WHOs. This study protocol for the randomised comparative functional study of the wrist/hand with and without WHOs was reported following the revised Standards for Quality Improvement Reporting Excellence 2.0 (26).

The WHOs’ effectiveness was assessed by comparing the maximum active range of wrist motion (from flexion to extension, and from ulnar to radial deviation); maximum grip strength; and the wrist position at maximum grip strength. All these tasks were performed by the participants with and without the WHOs.

### Subjects

Ten healthy right-handed (confirmed by the Edinburgh handedness test) female participants, aged (36 ± 10.8) years old, were recruited. Healthy participants were chosen instead of people with an underlying condition as pain can be a limiting factor to wrist ROM and grip strength, and it was important that all measured effects during testing were attributable directly to orthotic influence. Female participants were selected as RA is more prevalent in this group and hand strength has been reported to have a curvilinear relationship with age (27, 28), and is largely stable between 20 and 50 years (27). Moreover, as musculoskeletal conditions can negatively impact on upper limb strength and the upper-body strength of healthy female subjects has been shown to be 40–70% less than male equivalents (29), it was considered that the upper limb strength of the healthy female participants would adequately represent the upper limit of deforming force that could be applied to the WHO by either gender when presenting with wrist/hand dysfunction.

Exclusion criteria included subjects undertaking upper body/limb training during the test period; any musculoskeletal or neurological disorder affecting the upper limb; any injury affecting the hand, wrist and/or arm; and any previous upper limb surgery.

### Design

Existing literature often shows a lack of consistency in both conducting and reporting testing protocols, with an inadequate description of the interventions tested, and little attempt to quantify the degree of motion restriction as a result of the intervention. Therefore, a controlled, repeatable study was developed to evaluate the performance of ten commercially available prefabricated WHOs. This research study ran over a period of 10 weeks, with each participant testing a different WHO each week. The order of the WHO was computer-randomised for every participant.

Height, weight and maximum grip strength of each participant were recorded at the beginning of the 10 week period as there is a reported correlation between these parameters (30–32). Subsequently, participants’ weight was tracked throughout the test period to keep a strict control in the correlation between grip strength and weight (33) aiming to identify any confounding effect on the results.

### Hardware and Configuration

Two electro-goniometers (*SG65,* Biometrics Ltd.) were used to measure wrist flexion/extension and ulnar/radial deviation angles. These sensors were attached with double-sided medical grade tape, and further secured with an elasticated stockinet to minimise artefact movement. To minimise positional uncertainties when placing the electro-goniometers, each unit was checked using a traditional mechanical plastic goniometer and the unit was zeroed accordingly. To measure grip strength, a dynamometer (*G200,* Biometrics Ltd.) set to second handle position, was used. Of importance, the same researcher was responsible for fitting the sensors during all testing sessions and a rigorous protocol for sensor placement and calibration was used which ensured consistency and repeatability throughout the period of testing.

### Weekly Testing Protocol

**Figure 1:**
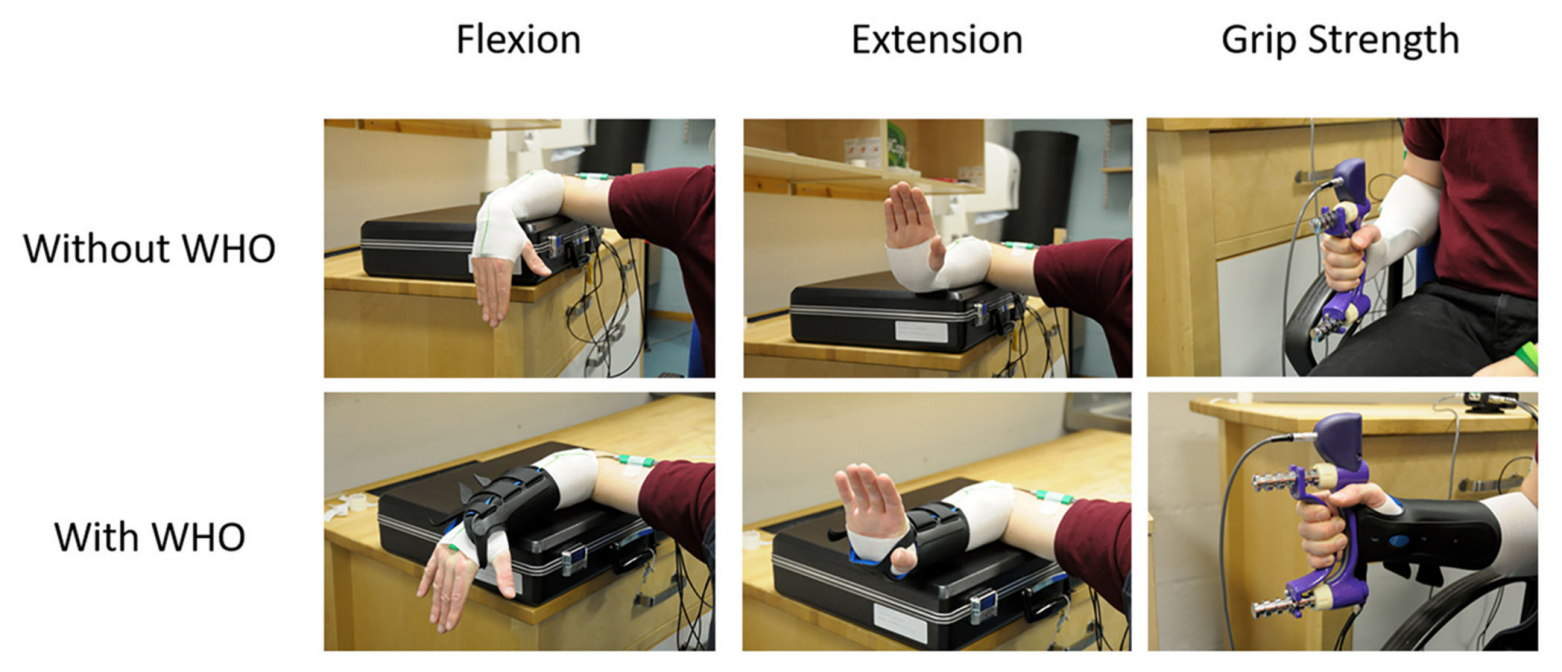
Typical example of the forearm supported on a table with the wrist and hand unsupported allowing them freedom in movement for wrist flexion and extension. Additionally, a typical example of seating alignment during a test for grip strength test. Elbow flexed at 90° with the forearm neutrally rotated with the thumb pointing upwards.

For both the ROM and grip strength tests each participant was seated on a height adjustable chair with knees and hips flexed to 90° and feet flat on the floor. To measure the wrist ROM with and without WHO, the right shoulder was abducted to 90° and neutrally rotated, elbow flexed at 90°, with the forearm supported on a table with the wrist and hand unsupported allowing the wrist to freely flex or extend (Figure 1). The ROM test protocol involved data acquisition during: (i) resting position (with WHO only); (ii) maximum wrist flexion; (iii) maximum wrist extension; (iv) maximum radial deviation; and (v) maximum ulnar deviation. A neutral wrist position was adopted between these movements under both conditions (with and without WHO). Participants were given verbal cues prior to each test and during each exertion they were encouraged to move through their maximum arc of motion.

For power grip strength tests, the right shoulder was adducted to a neutral and relaxed position, elbow flexed at 90° with the forearm in a neutrally rotated position and the thumb pointing upwards (34) (Figure 3). The wrist was in neutral radioulnar deviation with the fingers extended. Once in the starting position, the dynamometer was handed to the participant who was encouraged to sustain a maximum grip force for a period of 3 s (35–38). The position of the wrist in two anatomical planes when maximum grip strength was attained was also recorded to ascertain whether any restrictions in wrist motion with orthotic use could account for reduction in grip strength, if present. Participants were given verbal cues prior to each test and during each exertion were verbally encouraged to achieve maximum grip strength.

The ROM and grip strength protocols were repeated three times (35–38) with and without WHO, allowing for a 2 min rest period between repetitions to avoid participant’s fatigue.

One week between tests was considered an adequate resting period with minimal memory effect, yet short enough to avoid changes in power grip strength over the complete test period (39). Participants were instructed not to change their usual activities throughout the period of testing. The order of the ten WHOs was randomly allocated for each participant, as well as the order of wear/no wear (with/without WHO) at each test session. Each WHO was fitted to each participant according to the manufacturer’s guidelines by the same expert team, comprising of an orthotist and sports engineer throughout the duration of the study to ensure consistency and to ensure the optimal fit of each orthosis was achieved. The ten prefabricated WHOs that were selected for testing (Table 1), reflect variations in the commercially available designs with regards to geometry, materials of construction and fastenings. Although some of the WHOs tested had a removable aluminium volar bar, there was no bending of this to alter the alignment of the wrist section of the device prior to fitting, as a commissioned qualitative study undertaken by the researchers for Vs. Arthritis UK, has indicated that the volar bar is infrequently adjusted. The angle of this bar was measured before and after testing to cheque for any deformation of it as a result of activity.

### Outcomes of Interest

The functional characteristics of a well-designed WHO for many people presenting with wrist dysfunction are the prevention of wrist flexion from the optimal defined functional resting position (between 10 and 15° wrist extension and defined as 100% restriction relative to the maximum flexion without WHO), while maintaining grip strength (0% restriction relative to maximum grip strength without WHO). As such, different outcome measures were obtained from the two tests performed (i.e., ROM and grip strength).

### Statistical Analysis

#### Range of Motion

The effect that each WHO had on ROM was measured and compared to without WHO measures collected on the same testing day. The mean values and standard deviations (S.D.) across movement repetitions, with and without WHO, were measured from each participant and calculated when the wrist was:

a) at maximum extension (3 events);
b) at the resting position (9 events);
c) at its maximum flexion (3 events);
d) at maximum radial deviation (3 events); and
e) at maximum ulnar deviation (3 events).

For the ROM tests, the primary outcome measure was the resting position adopted in relation to the defined optimal position of 10 to 15°; and the restriction that each WHO had on flexion, extension, radial and ulnar deviation across the participants.

Wrist extension of 10 to 20° has been described as the optimal position for those users presenting with synovitis (3) as this reduces stress on peri-articular structures, the joint capsule and the synovial lining (40), whilst also optimising efficiency of the flexor muscles. However, the position should also reflect the best position for pain relief for the individual (39). There is lack of consensus regarding the exact position of the wrist in the sagittal plane to relieve symptoms associated with carpal tunnel syndrome (often present in those with RA), which varies from slight flexion (41), to a range of 10–15°extension (3) or neutral (42).

If the resting position is to be maintained during ADLs, and any propensity to wrist radial deviation is addressed, then the WHO should prevent motion beyond the defined resting position. Therefore, the restriction percentage for each movement (%R_movement_; Equation 1) was obtained for each participant by calculating the %R for each movement, and the overall means were obtained across the sampled population for each WHO, and across WHOs for each participant.

(1) $$\%R_{movement}=\left[100-\frac{\left(Maximum\;Movement\;with\;WHO\times 100\right)}{Maximum\;Movement\;with\;outWHO}\right]$$

As one aim of this study was to compare the performance of WHOs prescribed for RA management, the effectiveness that each WHO had in restricting flexion movement was quantified, and 1-sample t-test (α = 0.05) was used to measure the statistical difference between 0° (test mean, mu) and the measured maximum mean flexion per WHO. Similarly, the mean values at rest (as defined between 10 and 15° of extension) were obtained and a 1-sample t-test (α = 0.05) was used with mu = 10° and mu= 15° to obtain the statistical difference, if any, between each mu and the mean values at rest for each WHO. Finally, the maximum mean flexion and maximum mean extension were obtained and compared with and without WHO, using paired-sample *t*-tests (**α** = 0.05). For extension, radial, and ulnar deviation, the mean and S.D. were obtained and the %R_movement_ were calculated. Wilcoxon signed-rank tests were used to assess differences for non-parametric data.

#### Grip Strength

For the maximum grip strength test, the outcome measure of interest was the maximum peak force exerted and the adopted wrist position before exertion of force and when the maximum grip strength was achieved. The mean values and S.D. for the three maximum grip strength repetitions were obtained with and without WHO. The impact on grip strength was obtained by comparing the WHO and the without WHO condition using a paired-sample *t*-tests (α = 0.05). Additionally, the motion of the wrist was captured and compared between conditions to identify any wrist motion compensation strategies adopted by participants to attain maximum grip strength while wearing each WHO. These were assessed using paired-sample T-tests. Finally, the adopted position of the wrist before exertion of force, and while achieving a maximum grip strength was assessed and compared individually per participant with and without WHO.

## Results

A total of two participants missed each a single week of testing which corresponded to WHO#1 and #9 which resulted on the acquisition of datasets from 9 participants for each of these orthoses.

### Range of Motion

The resulting mean (S.D.) with and without WHO for the resting position and the resulting %R_flexion_ and %R_extension_ across participants is summarised in Table 2.

**Figure 2:**
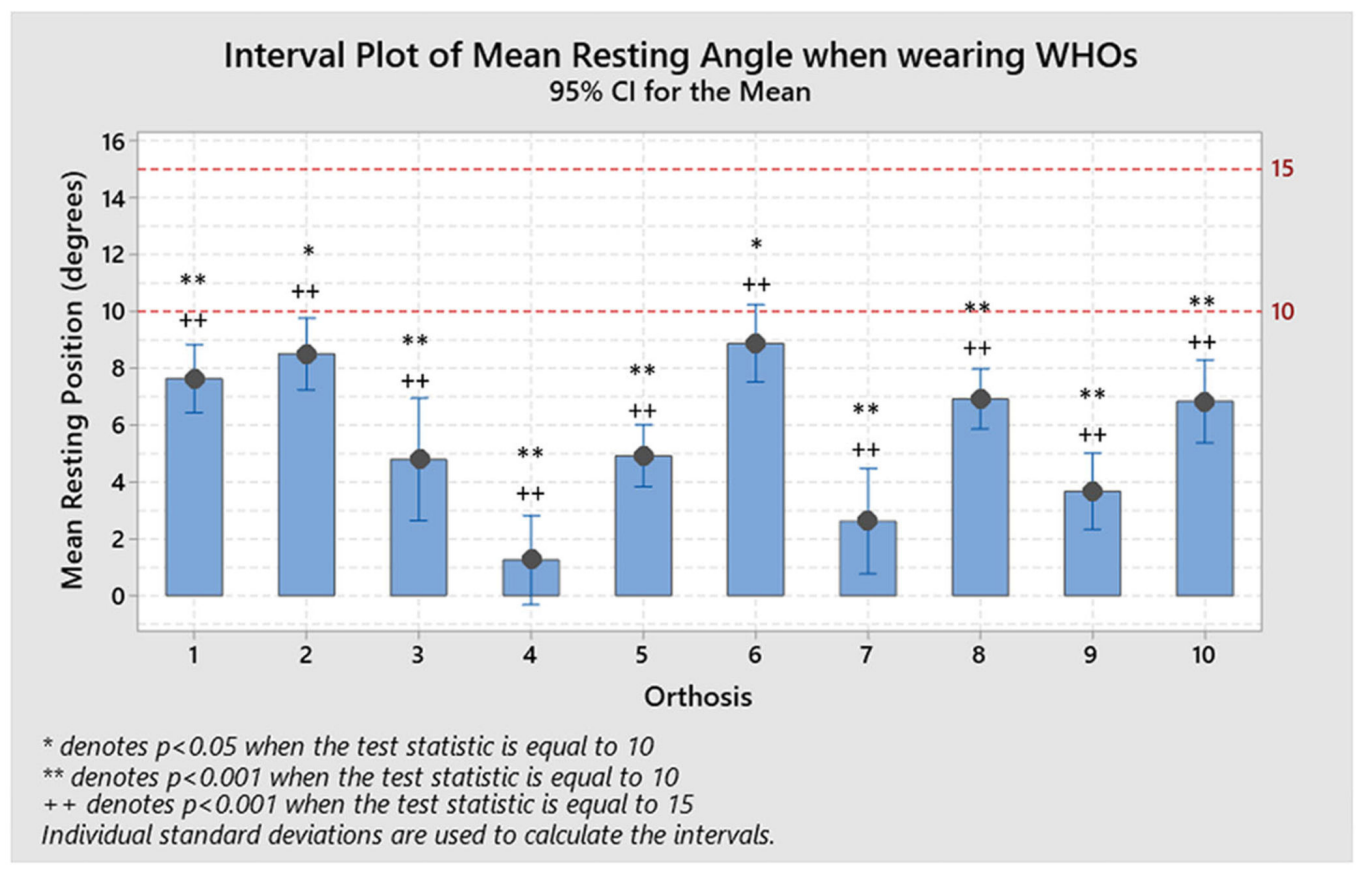
Resulting positions at rest for each WHO across the ten participants and compared to each recommended resting value of 15 and 10°. No orthosis performed as desired, with all ofthem being substantially and statistically different to the desired resting range (1-sample *t*-test, *p < 0.05 for mu = 10, **p < 0.001 for mu = 10, +*p* < 0.05 for mu = 15, and ++*p* < 0.001 for mu = 15).

The resulting resting positions across participants, as shown in Table 2, with WHO ranged from 1.3° extension (± 6.1°) for orthosis #4, to 8.9° extension (± 5.1°) for orthosis #6. All orthoses positioned the wrist in a degree of extension at rest (1-sample *T*-tests; mu = 10 and 15), but none were inside the desired prescribed range of (10–15)° extension (Figure 2).

**Figure 3:**
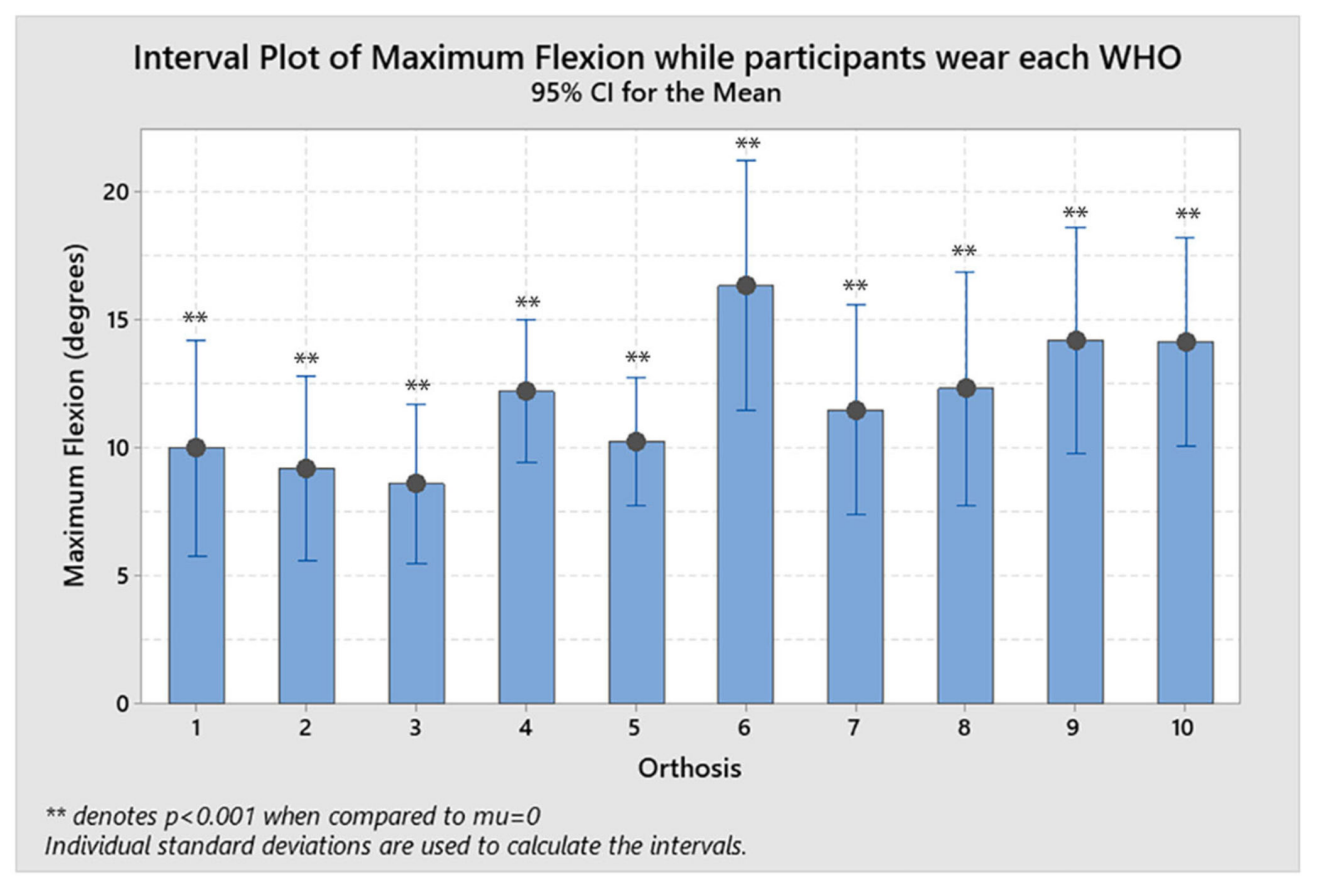
Resulting flexion allowed by each WHO with the statistical significance when a 1-sample *T*-test was performed with mu = 0 and is denoted as **p < 0.001.

The resulting flexion restriction (%R_flexion_; measured as a percentage in relation to the maximum arc of motion as shown in Equation 1) ranged from best reduction with a mean of 86.7% (± 14.1%) for orthosis #3, to 73.3% (± 19.0%) for orthosis #6 as the least restrictive (Table 2). Each %R_flexion_ was compared statistically to 0 with no WHO consistently preventing the wrist from moving into flexion (mu = 0, 1-sample T-test, *p* < 0.001; Figure 3).

The resulting extension restriction (%R_extension_) ranged from a mean of 76.7% (± 17.6%) for orthosis #4 to 33.2% (±28.4%) for orthosis #6 (Table 2).

**Figure 4:**
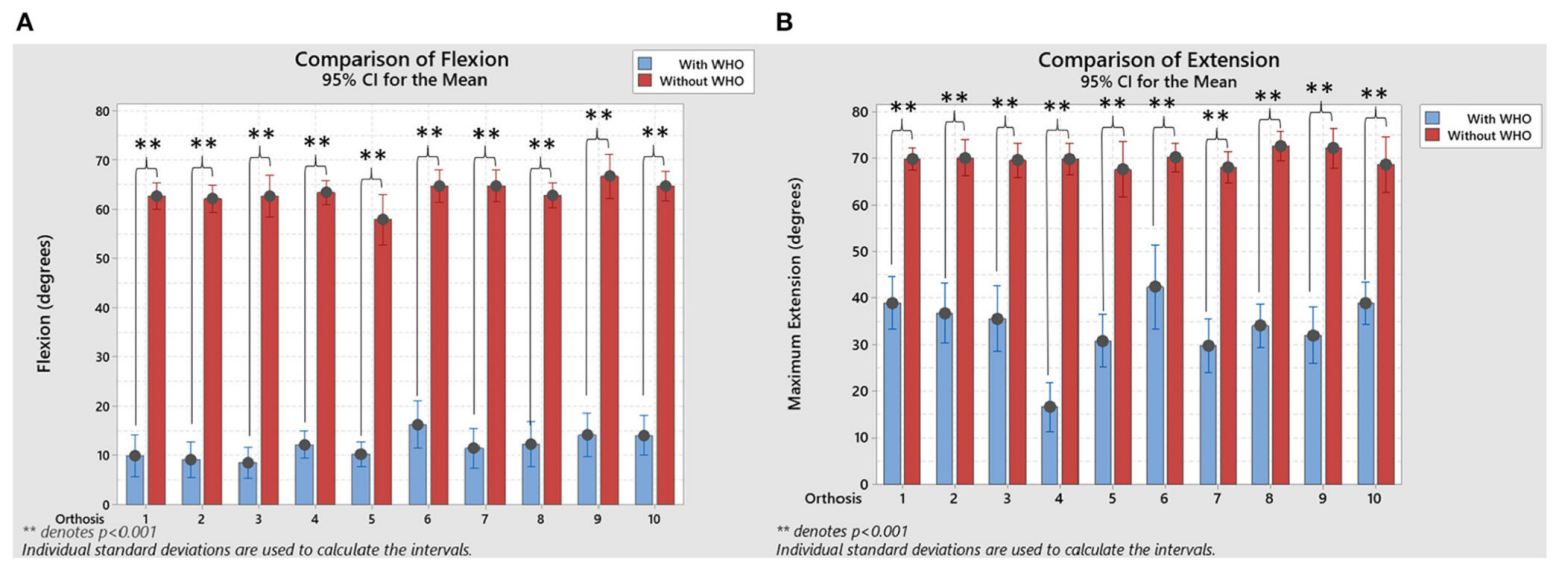
**(A)** Resulting flexion comparison, with and without WHO, with statistical significance when a paired-sample *T*-tests were used to compare between them. **(B)** Resulting extension comparison, with and without WHO, with statistical significance when a paired-sample *T*-tests were used to compare between them. **p < 0.001.

When flexion and extension movements were compared between with and without WHO, statistically significant differences were seen (paired-sample *t*-tests, p < 0.001; Figure 4).

The resulting position at rest, and maximum radial and ulnar deviation, with and without WHO, and their respective %R is shown in Table 3.

As seen in Table 3, the mean resting positions across all WHOs, ranged from 5.2° (± 4.9°) ulnar deviation for orthosis #9, to 9.2° (± 5.7°) radial deviation for orthosis #3, with all WHOs except one (#9) positioning the wrist in radial deviation at rest.

**Figure 5:**
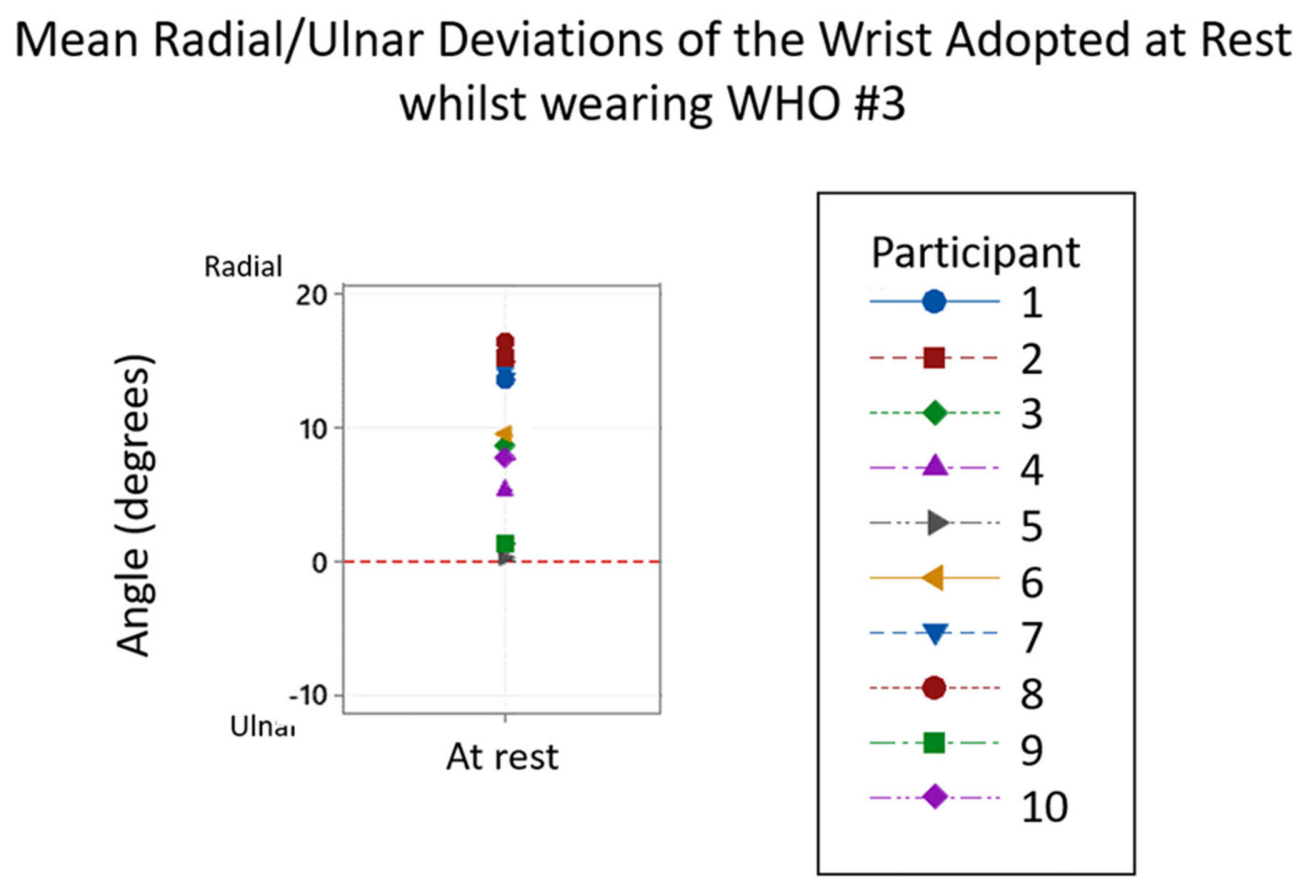
Typical example of the ulnar and radial deviations of the wrist adopted at rest whilst wearing Orthosis #3. From the 10 participants, all adopted a position of ulnar deviation at rest.

Given the wide distribution in the data, the positions of radial and ulnar deviation were compared across participants for each individual WHO as presented in Table 4, with a typical example of a WHO chosen at random (#3) shown in Figure 5.

**Figure 6:**
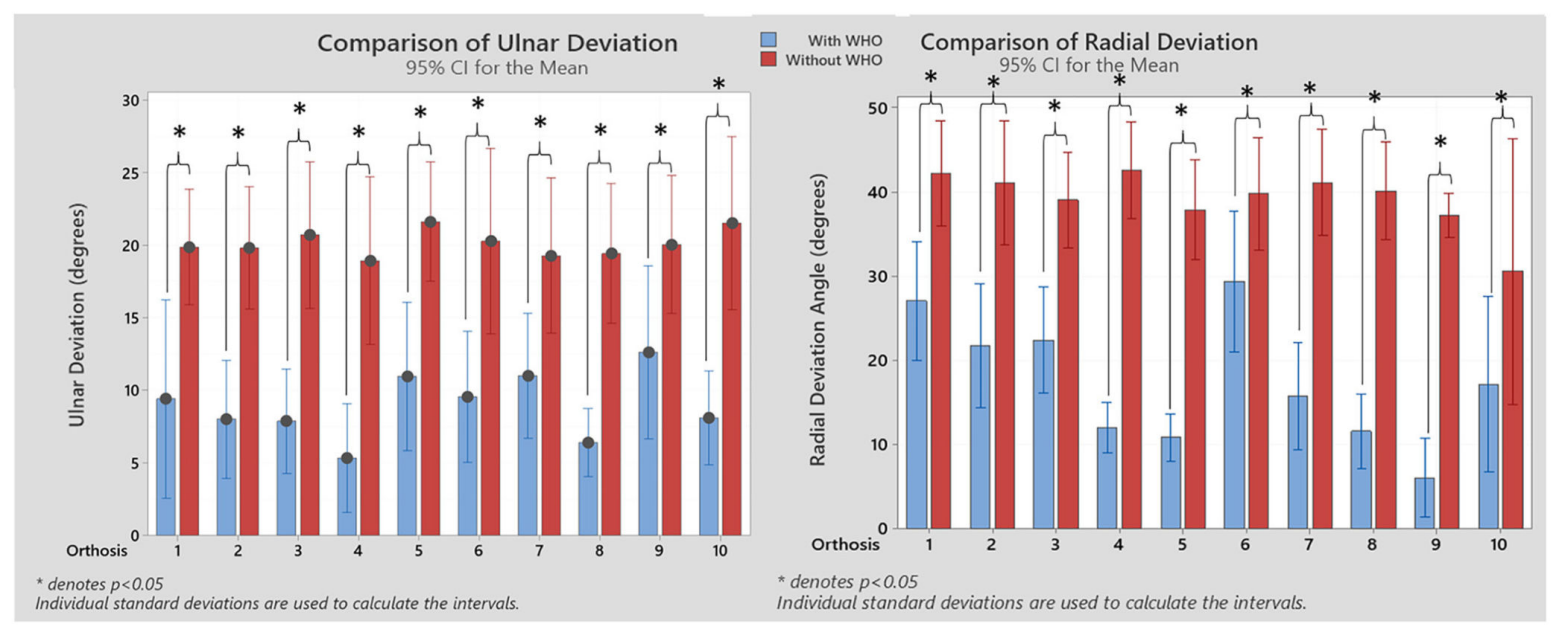
Resulting ulnar and radial deviation angles with and without WHO. Statistical significant differences between conditions are shown (Wilcoxon signed-rank I tests). *p < 0.05.

With regards to motion restriction, all WHOs had a certain degree of radial deviation restriction, with orthosis #9 being the most restrictive with a mean average %R_radialdeviation_ of 83.5 % (± 16.1 %) with least restrictive being orthosis #6 with 27.7% (± 29.7 %). When the ulnar deviation was assessed, orthosis #4 was the most restrictive with a mean %R_ulnardeviation_ of 108.6 % (± 40.6 %), and orthosis #7 being the least restrictive orthosis with 35.3 % (± 44.5 %). The resulting radial and ulnar comparisons between with and without WHO across participants is shown in Figure 6.

### Grip Strength

In terms of participants’ weight, there was no more than a 2 kg variation across the testing period for any participant, as such, there was no need to factor this into the grip strength results.

The resulting mean (S.D.) maximum grip strength with and without WHO, its corresponding %R and wrist position at maximum grip strength is summarised in Table 5.

**Figure 7:**
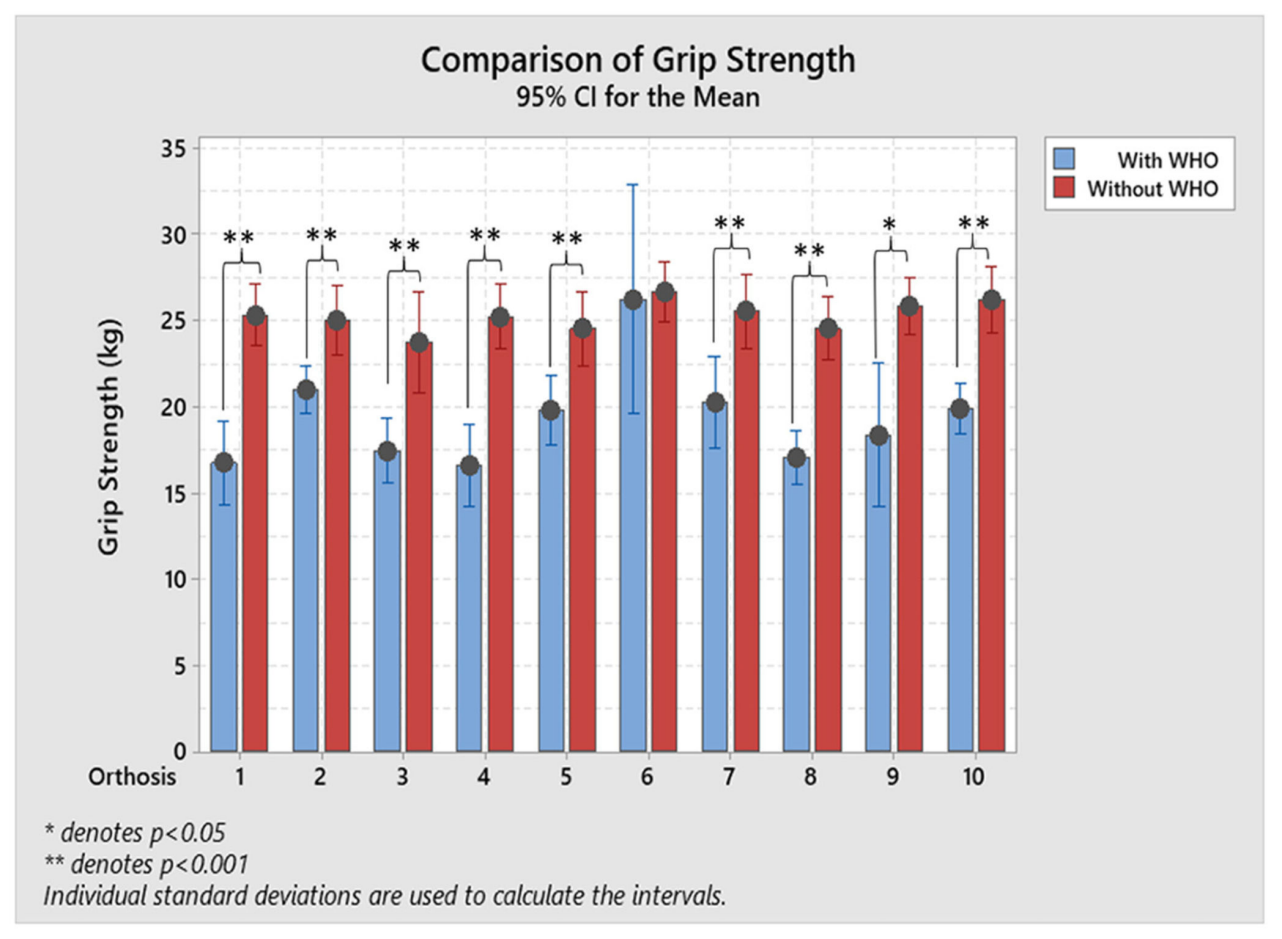
Resulting maximum mean grip strength with and without. Paired-sample *T*-tests were used to assess the statistically significant reductions on grip strength with *p < 0.05, and **p < 0.001.

As shown in the results when no WHO was worn, maximum power grip was achieved with the wrist in a position of extension ranging from (26.8°± 12.4°) to (33.9°± 7.6°). Results show that grip strength exerted by all participants for each WHO had a statistically significant decrease when compared to not wearing any WHO, with exception of orthosis #6 (paired-sample T-tests, p < 0.001, Figure 7). The mean average %R _gripstrength_ ranged from 1.7 % (±13.4 %) with orthosis #6 to 34.2 % (±10.6 %) with orthosis #4.

As shown in Table 5, these results show that the wrist position in both the sagittal and coronal planes when maximum grip strength was attained significantly changed when a WHO was worn. In all cases wrist extension and ulnar deviation reduced, while in some cases the wrist moved into a position of flexion and radial deviation.

**Figure 8:**
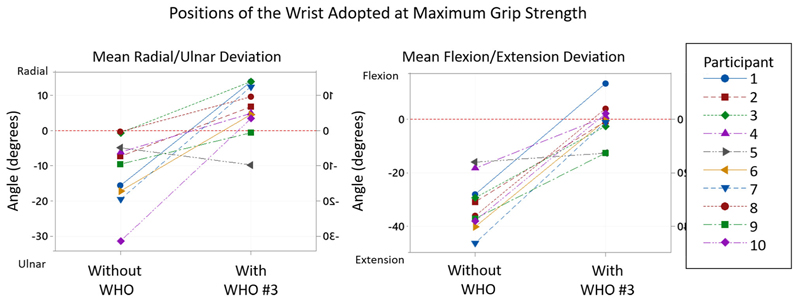
Typical example of the positions of the wrist adopted at maximum grip strength without WHO and with Orthosis #3. From the 10 participants, whilst the maximum grip strength was achieved, all adopted a position of ulnar deviation and extension of the wrist without WHO, whilst 8 moved into radial deviation and 3 shifted to a flexed position whilst wearing orthosis #3.

In order to better understand the change in wrist position, each test scenario can be considered. This information is presented in Table 6 and a specific example of a WHO (#3) is shown in Figure 8.

In the coronal place, ROM tests demonstrate that except for #10, WHOs reduce ulnar deviation to less than what is attained at mean maximum grip strength with no WHO. However, all WHOs except #4 which blocks ulnar deviation do still allow ulnar deviation to occur, yet this arc of motion was not utilised during grip strength tests, with a position of radial deviation mostly attained.

## Discussion

When a prefabricated WHO is prescribed, it is often selected from catalogues which state the health conditions for which the WHO can be used to “immobilize,” “provide stability,” or “support” the wrist. While the meaning of immobilisation is clearly understood, there is a lack of clarity regarding what a prescribing clinician should understand by “stability and support.” Also, there is no attempt to describe whether this purported function such as immobilisation while the wrist is at rest, is maintained during activity.

For this reason, the ROM tests in this study challenge the assumptions that WHOs hold the wrist in an acceptable position to facilitate WHO users to undertake ADLs with reduced pain and a stable wrist. Importantly and to improve prescription practises, clinicians need to appreciate the ways in which the design features of a WHO and its fit impact on functionality. The analysed data demonstrates that while there may be some reduction in flexion, extension, radial, and ulnar deviation, none of the WHOs successfully and consistently immobilised the wrist and crucially, none prevented movement into flexion. Allowing movement into flexion will not only negatively impact on synovitis but will consequently adversely affect the patient’s hand and wrist function. Additionally, the results show that the WHOs had a negative impact on the participants’ grip strength with their wrist adopting an abnormal positions. This reduction in grip strength will render some ADLs difficult to do and others which may involve carrying heavy or hot objects and substances dangerous to attempt. If adequate grip strength can only be achieved with the adoption of abnormal wrist positions, this warrants concern as many users have underlying conditions which are degenerative and progressive in nature.

### Interpretation

#### Range of Motion

The resulting resting positions across participants, as shown in Table 2, with WHO ranged from 1.3° extension (± 6.1°) for orthosis #4, to 8.9° extension (± 5.1°) for orthosis #6, with none positioning the wrist within the desired prescribed range of (10–15)° extension. As the WHOs were fitted as supplied by manufacturers, with no contouring of the volar bar to alter the alignment of the wrist section, these results emphasise that clinicians must not assume that manufacturers provide these WHOs with a volar bar contoured to hold the wrist in a suitable angle at rest and should be adjusted if possible.

The resulting flexion restriction ranged from best reduction with a mean of 86.7% (± 14.1%) for orthosis #3, to 73.3% (± 19.0%) for orthosis #6, as the worst, with no orthosis found to consistently prevent the wrist from moving into flexion, with all WHOs being statistically significantly different to 0 (mu = 0, 1-sample *T*-test, *p* < 0.001).

When the results across all ten WHOs are considered, aspects which may influence flexion control can be compared. There is some evidence in the results which suggest that there are many interrelated factors including the length of the orthosis, quality of fit, materials of construction, and the design and location of fastenings which influence motion control.

**Figure 9:**
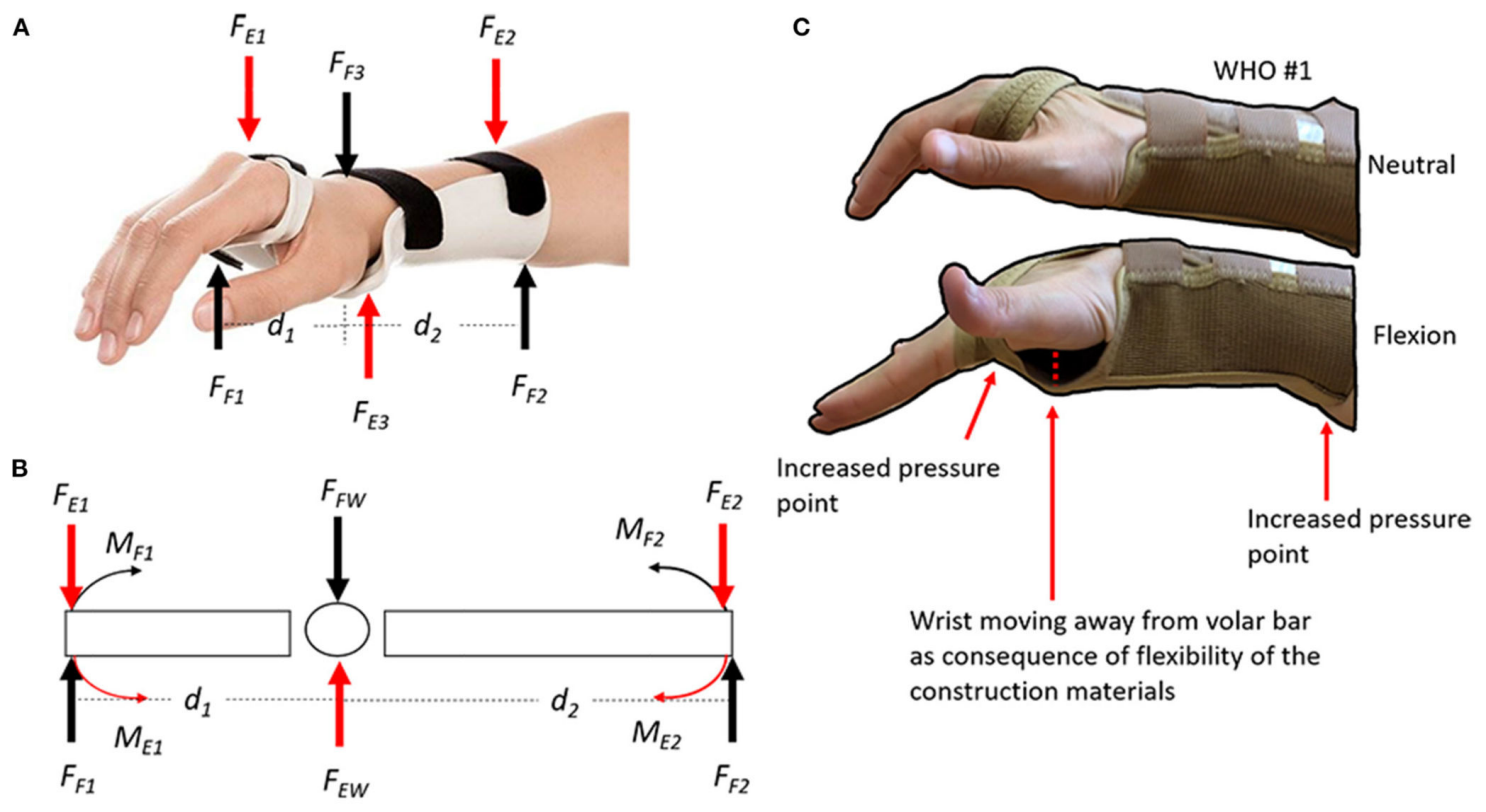
**(A)** Wrist flexion control forces (FF1, FF2, and FF3) and extension control forces (FE1, FE2, and FE3) from the WHO; **(B)** Free body diagram of flexion and extension forces. The magnitude of the moment of each force can be calculated using the equation *moment of a force* (M) = force* distance. The longer the distance the bigger the angular moment; and **(C)** Demonstration of flexion control forces when using WHO #3, which are typically reliant primarily on a volar bar (aluminium or plastic) which is often housed in a fabric pocket extending from the palm to the forearm. While this volar bar may be sufficiently stiff in terms of Youngs Modulus of Elasticity to provide motion control, it is often held in position by a flexible interface which compromises its functionality. During flexion, the volar bar will create an increased pressure point at the palm of the hand and at the posterior side of the forearm. Additionally, the wrist moves away from the volar bar because of the flexibility in the WHOs’ construction material (often neoprene or two-way stretch fabric).

The flexion control afforded by many WHOs is typically reliant primarily on the volar bar (aluminium or plastic) which is often housed in a fabric pocket extending from the palm to the forearm. While this volar bar may be sufficiently stiff in terms of Youngs Modulus of Elasticity to provide motion control, it is held in position by a flexible interface which compromises its functionality (Figure 9C).

Typically, those longer WHOs (23 cm in length, #1– 3) performed better, while those shorter WHOs (18 and 20 cm) performed less well. This is unsurprising when basic biomechanics are considered. The flexion moment generated during the flexion test must be resisted by the orthosis (Moment = force × distance), as such, the longer the lever arm, the more effective the orthosis will be in resisting these forces (Figures 9A,B).

Interestingly, orthoses #7 and #4, which were 25 cm in length, performed worse than those which were 23 cm in length. These orthoses fitted participants less well due to their construction. For example, orthosis #4 was a high durometer silicone WHO which completely circumferentially contained the forearm, wrist, and hand stopping just proximal to the metacarpal-phalangeal joints. The lack of adjustability in this WHO to optimise fit is likely to have adversely affected motion control. The only difference between orthosis #2 and #1 was the material of construction as both were the same length and had identical aluminium volar bars. Orthosis #2 made from neoprene (synthetic rubber) performed better than #1 which was made from knitted two-way stretch elasticated material. While the mechanical tensile properties of these fabric materials have not yet been measured using an INSTRON tensile tester, it was clear during testing that the knitted elasticated fabric exhibited significantly more stretch. While material of construction might relate to WHO functionality, the other properties of these materials must be considered. For example, while neoprene is resistant to water, oils, and chemicals all of which a user may come into contact with during use, it is has poor moisture wicking properties, poor breathability and retains heat, all of which are undesirable features to a user. Orthosis #3 and #1 were identical except for the presence of an additional circumferential wrist strap present in #3, as such, it is clear from the results that the presence of this additional strap provides enhanced flexion control. Indeed, its presence seems to be as important as the material from which the orthosis is made. If we compare the improved flexion results from orthosis #3 compared to #2 made from knitted elasticated fabric and neoprene, respectively, the additional wrist strap is clearly beneficial.

Extension control across all WHOs was much poorer than flexion control with an optimal %R_extension_ of 76.7 % (± 17.6%) for orthosis #4 (25 cm silicone WHO) with the next best significantly less at 56.1 % (±21.1%) for #7 (25 cm neoprene). This was unsurprising as orthosis #4 which fully encompassed the hand, wrist, and forearm with high durometer silicone was the most rigid in the dorsal aspect. None of the other orthoses were sufficiently stiff in the necessary areas on the dorsal surface for optimally controlling extension, with all the tested WHOs having a statistically significant decrease in wrist extension.

Those orthoses #7, #9, #8, and #5 which were the next most effective, each had flexible plastic struts positioned on the dorsal surface for strap attachment which positively impacted on the ability of the orthosis to control extension. Orthosis #7 which performed better than #5 were identical other than in length (25 and 20 cm respectively), once again highlighting that increased length positively influences motion control. In addition to having dorsal plastic stays for strap attachment, orthosis #8, although only 20 cm in length, is made from fabric with minimal stretch and a broad circumferential wrist strap, the combination of which would assist with extension control. In summary, none of the tested orthoses limit extension across the majority of participants within the desired range of 10 to 15° extension.

It was important to ascertain which of the orthoses had design features to resist radial deviation of the wrist so that ulnar drift of the fingers associated with RA could be addressed. Although their use is often recommended for this condition, it should be considered that perhaps none of these orthoses have been designed specifically for the RA wrist and therefore consideration of this aspect of deformity may not have been adequately considered. Indeed, none of the WHOs had specific areas of stiffness which would be required to control radial deviation.

As seen in Table 3, at rest, other than with #9, the wrist mean angle was one of radial deviation, not neutral or ulnar deviation as would be required. None of the WHOs tested have design features which would allow this angle to be adj usted at fitting. The mean resting positions across all WHOs, range from 5.2° (± 4.9°) ulnar deviation for orthosis #9, to 9.2° (± 5.7°) radial deviation for orthosis #3.

With regards to motion restriction, all WHOs had a certain degree of radial deviation restriction, with orthosis #9 being the most restrictive with a mean average %R_radialdeviation_ of 83.5 % (± 16.1 %) with least restrictive being orthosis #6 with 27.7 % (± 29.7 %). Only four WHOs, #9, #8, #4, #5, could be considered to provide substantial restriction of radial deviation ranging from 83.5 % to 70.7%, respectively. Unsurprisingly, none of these four WHOs were made from two-way stretch fabric which as previously discussed has more stretch and in the absence of design features to control radial deviation would not provide adequate motion restriction. Orthosis #9 which was the only WHO with a resting angle of ulnar deviation was the most restrictive with a mean average %R_radialdeviation_ of 83.5% (± 16.1%). Orthosis #8 is a simple ‘slip-on’ design and incorporates a unique fastening system which tensions the orthosis with one strap closure which is excellent for patients with limited dexterity. Due to the orientation of the closure system and direction of pull from the radial to ulnar aspect, it is entirely possible that this provides enhanced radial deviation control. Orthoses #8 and #5 have plastic stays for strap attachment so although not perhaps intended for control of radial deviation it is entirely likely that these provided some reinforcement of the fabric to provide enhanced motion control. Similar to the results found in the sagittal plane, it is therefore acknowledged that strap location and tensioning of the straps may influence the resting position and motion restriction. Orthosis #4 provided a mean average %R_radialdeviation_ of 70.7% (± 10.7%) as it circumferentially encapsulates the hand, wrist and forearm with high durometer silicone, providing stiffness in key areas. Due to its design, it might be expected that it would be the most successful in providing this motion control, however the results for this orthosis shows it was not consistent in positioning the wrist at rest and providing motion control across participants. As this orthosis has least adjustability, it highlights the issue pertaining to achieving an optimal fit which has a relationship with functionality. Furthermore, the most restricting orthosis when ulnar deviation was assessed, was orthosis #4, with 108.6% (± 40.6%) restriction, and orthosis #7 being the least restrictive orthosis with 35.3% (± 44.5%) of ulnar restriction.

In summary, if the orthosis is to position the wrist and limit motion appropriately, it needs to be sufficiently stiff in key areas for motion control, but flexible and/or adjustable in other areas to optimise fit. The location of the stiff areas must not be dependent on the quality of fit, i.e., the donning and fastening of the orthosis should not cause the parts to become poorly positioned or misaligned if optimal motion control is to be achieved.

#### Grip Strength

As shown in the results when no WHO was worn, and as evidenced by the literature (6, 43), maximum power grip was achieved with the wrist in a position of extension ranging from (26.8 ± 12.4°) to (33.9 ± 7.6°). However, positioning the wrist in this amount of extension with a WHO would interfere with many ADLs. If orthoses can prevent motion of the wrist from a position of [10–15°] of extension, into aless extended position, this would also address clinical issues of synovitis and/or carpal tunnel syndrome while better allowing ADLs to be undertaken. Lee and Sechachalam investigated the impact of sagittal plane wrist position on mean grip strength (7). Wrist position was controlled by an orthosis and while mean grip strength of the dominant hand was reduced by 6% and up to 43%, there was not statistical difference in mean grip strength between wrist positions of 15 and 30°, which was significantly higher than that measured at 0° and in flexion. Although a little controversial in the literature, there is strong evidence that up to 15° of ulnar deviation is required for maximum grip strength (6,8) which is demonstrated in the presented results when no WHO was worn. In a patient with RA, a position of [5–10°] of ulnar deviation is preferable to counterbalance the zigzag deformity of radial deviation of the wrist with ulnar drift of the fingers. This highlights that while wearing a WHO, perhaps there is need to block radial deviation and, when appropriate, allow some ulnar deviation. A reduced position of extension and ulnar deviation may negatively impact grip strength as reported by other authors. O’Driscoll et al. reported that a position of 15° extension with 5° ulnar deviation results in 66% to 83% of normal grip strength (43).

The use of a WHO has also been reported in the literature to negatively impact on grip strength, particularly over the shorter term but improve grip strength over a longer period (14–16, 22, 24–26). However, it is important to appreciate that in these studies this typical trajectory of reduced strength followed by increased strength with splint wearing over time may reflect a forced reduction in loading at the wrist joint with subsequent settling of the inflammatory process and reduction in pain followed by improved motor performance. However, as this study is measuring the immediate impact on grip strength on healthy participants, an initial reduction in grip strength would not necessarily increase over a longer period of time. Results show that grip strength exerted by all participants for each WHO had a statistically significant decrease when compared to not wearing any WHO, with exception of orthosis #6. The mean average %R_gripstrength_ ranged from 1.7% (±13.4%) with orthosis #6 (18 cm knitted elasticated fabric with volar bar, Table 1) to 34.2% (±10.6%) with orthosis #4.

The results in Table 6 show that the wrist position in both the sagittal and coronal planes when maximum grip strength was attained significantly changed when a WHO was worn. In all cases wrist extension and ulnar deviation reduced, while in some cases the wrist moved into a position of flexion and radial deviation.

There is therefore a need to further explore the wrist position adopted in both sagittal and coronal planes when maximum grip strength was reached, with and without WHO, to better understand whether motion restriction as a result of WHO wear is responsible for the reduction in grip strength. When considering motion control tests presented in Tables 2, 3, WHO #6 demonstrated least restriction of extension, but was one of the best at restricting ulnar deviation, respectively. Although this WHO has a metal volar bar in the palm of the hand which typically prohibits grip strength, the poor design characteristics of the WHO mean that participants maybe able to overcome any impact of it by adopting an alternative wrist and grip position to achieve good grip strength (as seen in Table 5). Cross-referencing of the results in Tables 2, 3, 5 allows this to be investigated. Other than orthosis #4, no orthosis was responsible for restricting wrist extension to a level which would impact at all on grip strength. Yet despite this, during grip strength tests wearing WHOs, the mean sagittal plane position attained at maximum grip strength ranged from wrist flexion of 0.7° (± 8.4°) to extension of 6.1° (± 13.3°). This suggests that due to the presence of the WHO and unyielding volar bar in the palm of the hand, participants had to adopt abnormal sagittal plane wrist positions to achieve maximum grip strength. Orthosis #4 motion control tests were shown to restrict extension to a mean angle of 16.6° (± 14.2°), less than the wrist extension angle of 32.2 ° (± 10.31°) when maximum grip strength was achieved with no WHO. However, when wearing this WHO during grip strength tests, a mean extension angle of only 0.78° (± 8.82°) was measured which also shows that other factors probably relating to the bulky palm section of the orthosis impacted the ability to achieve a normal power grip pattern. In the coronal plane, ROM tests demonstrate that except for #10, WHOs reduce ulnar deviation below what is attained at mean maximum grip strength with no WHO. However, all WHOs except #4 which blocks ulnar deviation do still allow ulnar deviation to occur, yet this arc of motion was not utilised during grip strength tests, with a position of radial deviation mostly attained.

In summary, the prefabricated WHOs tested cannot be recontoured to better reflect the shape of the transverse palmar arch. The poor fit of WHOs in this area means that not only is the transverse arch of the hand not optimally supported as required, but there is a direct negative impact on grip strength, as highlighted in the results. The poor contouring and unyielding nature of the WHO in the palmar section makes gripping of many objects (in this case the dynamometer handle) even more problematic and may force the user to adopt a different grip pattern and unusual wrist position. This is an aspect which can be related to previous studies investigating grip strength. Some investigators used a Jamar® dynamometer to measure grip strength while others used a more compliant device such as a vigorimeter (44). This could therefore suggest that a WHO may affect grip strength more when grasping an unyielding object than when grasping a compliant one. This area requires further research to explore different grip patterns during activities of daily living, as it is clearly of concern that globally, clinicians are prescribing WHOs which may encourage abnormal wrist positions to be adopted and potentially exacerbate pain.

#### Study Limitations

As patients with RA, and many other conditions, are likely to experience pain which may limit wrist motion and grip strength, healthy participants were recruited for testing so that the biomechanical efficacy of the WHOs could be tested without the influence of confounding factors.

The Biometrics DataLOG unit, goniometers and Jamar dynamometer were subject to accuracy uncertainties, with ±2° as listed in the specifications for the goniometers and a resolution of ±0.9 kg for the dynamometer (45). However, to minimise positional uncertainties when placing the electrogoniometers, each unit was checked using a traditional plastic mechanical goniometer.

An additional limitation may relate to the fit of the WHOs. Although participants were given a WHO appropriately sized for their wrist in line with the manufacturer’s sizing guidelines, this does not guarantee optimal fit. To minimise this, once fitted, and with appropriate tensioning of the straps, a further visual assessment of fit was made as is done in normal clinical practise. If this was deemed to be acceptable, testing was undertaken. Importantly, there are no known methods to accurately quantify the quality of fit of a WHO, hence errors in the results pertaining to fit could not be quantified.

As all WHOs were fitted by the research team, the best fit for each WHO was achieved, thus demonstrating the maximum potential efficacy of each WHO as a result of the design characteristics. However, in practise a WHO is fitted by individuals themselves who would be unlikely to consistently and repeatably achieve this optimal fit using their contralateral hand which may also present with dysfunction. As such this present research demonstrates the optimal efficacy of the WHOs which may not be achievable in the patient group when independently donned.

Finally, it should be acknowledged that a greater sample size would allow for additional biomechanical comparison of the WHOs. However, given the lack of data variability across the sampled participants during the high number of repetitions presented in this study, the authors would not expect a greater sample size of healthy participants to lead to different results.

#### Conclusions

This present work raises questions regarding the design of prefabricated WHOs which has essentially remained unchanged for several decades. Variations in the efficacy between the WHOs tested might be a contributing factor for the current conflicting evidence of efficacy and functionality for WHOs and suggests that there is the potential for re-design of WHOs to address the objectives of orthotic management. Appropriate design and positioning of straps can provide essential motion control forces, and therefore must not be considered as only a way of retaining the WHO on the arm. In this study, the WHOs were fitted by the same researcher to each participant to ensure optimal fit and function, but it must be recognised that most wearers don these devices independently. As such, their design, including the position and type of straps, has an impact on the user’s ability to put it on and fasten it using one hand only. The difficulty in achieving this, may have a direct impact on quality of fit, comfort and functionality of the orthosis and further research is required to investigate this.

To inform evidence-based practise, there is a current need for researchers working in this area to conduct testing using robust methodologies, and report consistently precise information about the study, including the methodology, participant characteristics and orthoses tested. This would make the research repeatable and clinically relevant, thereby positively impacting patient care. The authors recognise the requirement for further research into the use of “functional” pre-fabricated WHOs to evaluate the long-term impact of use on grip strength, grip endurance, and other parameters such as activities of daily living and pain (amongst many others). Importantly, the factors affecting user adherence must be fully considered. Research has shown that bulky, poorly fitting, uncomfortable WHOs that are easily soiled, negatively impact adherence. In addition, there are significant concerns regarding poor aesthetics, difficulty on putting on/taking off the WHO and the reliance on Velcro™ fastenings. WHO wearers indicate they wish to have devices which provide more support and reduce pain which can improve the ability to carry out ADLs (46). Only by addressing these and improving WHO functionality is there the potential to achieve positive impact on quality of life for wearer of these devices regardless of the underlying condition and health economics.

## Figures and Tables

**Table 1:** Range of commercially available wrist-hand orthoses used in this study showing their length (in cm), construction material, fastenings, type of volar bar, and presence of additional wrap around wrist strap.

ID	Length (cm)	Construction material and fastenings	Volar bar material	Wrist strap	Image
1	23	Two-way stretch fabric and Velcro^®^ fastenings	Aluminium	N	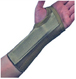
2	23	Neoprene with Velcro^®^ fastenings	Aluminium	N	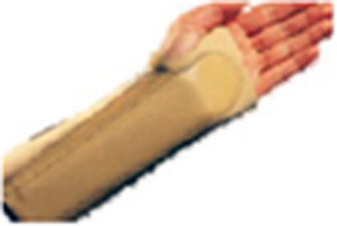
3	23	Two-way stretch fabric and Velcro^®^ fastenings	Aluminium	Y	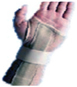
4	25	Silicone and Velcro^®^ fastenings	Plastic	N	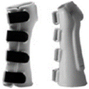
5	20	Neoprene with dorsal plastic stays and Velcro^®^ fastenings	Aluminium	N	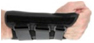
6	18	Two-way stretch fabric and Velcro^®^ fastenings	Aluminium	N	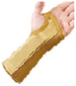
7	25	Neoprene with dorsal plastic stays and Velcro^®^ fastenings	Aluminium	N	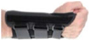
8	20	Fabric type, single lace, and/or Velcro^®^ fastenings	Aluminium	N	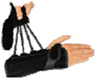
9	20	Neoprene with plastic pocket and Velcro^®^ fastenings	Aluminium	N	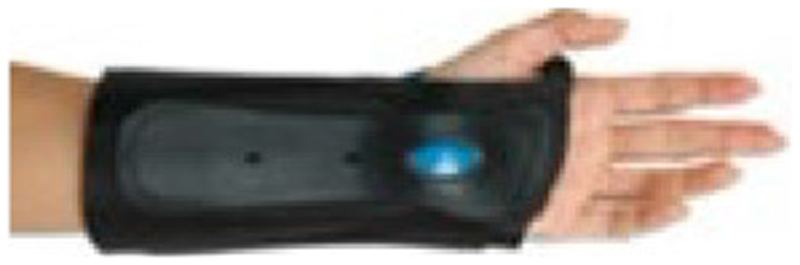
10	18	Neoprene and Velcro^®^ fastenings	Aluminium	N	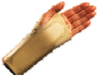

**Table 2:** Mean angles (S.D.) at resting position (with WHO only), wrist flexion and extension angles (S.D.) with and without WHO, and their %R as calculated with Equation 1.

WHO	Resting [°]	Maximum flexion [°]			Maximum extension [°]		
	Mean(S.D.)	Mean (S.D.)		%R Flexion	Mean (S.D.)		%R Extension
	With WHO	Without WHO	With WHO		Without WHO	With WHO	
1	-7.6	62.6	10.0	85.2	70.0	39.1	44.6
	(4.7)	(7.0)	(11.3)	(18.2)**	(6.4)	(15.2)	(19.9)**
2	-8.5	62.1	9.2	85.9	70.2	36.8	47.6
	(5.0)	(7.4)	(9.7)	(15.3)**	(10.5)	(17.2)	(22.5)**
3	-4.8	62.6	9.1	86.7	69.7	35.7	48.7
	(8.3)	(11.4)	(9.0)	(14.1)**	(10.0)	(19.0)	(25.3)**
4	-1.3	63.4	12.4	81.2	70.0	16.6	76.7
	(6.1)	(6.6)	(9.2)	(10.4)**	(8.9)	(14.2)	(17.6)**
5	-4.9	57.9	14.6	83.8	67.7	30.9	51.4
	(4.3)	(13.7)	(7.4)	(9.5)**	(15.9)	(15.0)	(25.1)**
6	-8.9	64.7	19.8	73.3	70.2	42.5	33.2
	(5.1)	(8.3)	(13.4)	(19.0)**	(7.7)	(24.3)	(28.4)**
7	-2.6	64.7	12.8	83.4	68.1	29.9	56.1
	(7.2)	(8.6)	(10.9)	(16.2)**	(9.2)	(15.2)	(21.1)**
8	-6.9	62.7	10.3	81.0	72.7	34.1	53.0
	(4.2)	(6.7)	(9.3)	(19.1)**	(8.7)	(12.6)	(16.5)**
9	-3.7	66.7	10.4	80.4	72.2	32.1	54.9
	(5.0)	(11.9)	(11.2)	(15.2)**	(11.6)	(15.3)	(22.4)**
10	-6.8	64.6	18.6	78.3	68.8	39.0	44.7
	(5.6)	(8.0)	(11.3)	(17.3)**	(16.0)	(12.3)	(18.4)**

Negative numbers imply the wrist is in extension. Paired-sample t-tests (α = 0.05) were applied to assess mean differences between with and without WHO. **p < 0.001.

**Table 3:** Mean angles (S.D.) at resting position, maximum radial and ulnar deviation with and without the orthosis, and their %R as calculated with Equation 1.

WHO	Resting [°]	Maximum ulnar deviation [°]			Maximum radial deviation [°]		
	Mean (S.D.)	Mean (S.D.)		%R ulnar deviation	Mean (S.D.)		%R radial deviation
	With WHO	Without WHO	With WHO		Without WHO	With WHO
1	6.0	19.9	9.4	52.2	42.2	27.1	35.6
	(6.4)	(5.2)	(8.4)	(43.2)*	(8.1)	(9.2)	(16.8)*
2	5.0	19.8	7.4	66.5	41.1	21.8	47.5
	(6.2)	(5.9)	(6.2)	(32.6)*	(10.3)	(10.3)	(22.8)*
3	9.2	20.7	6.7	77.3	39.0	22.4	43.3
	(5.7)	(7.1)	(6.3)	(44.8)*	(7.9)	(8.9)	(15.7)*
4	6.6	18.9	0.0	108.6	42.6	12.0	71.1
	(6.4)	(8.1)	(7.3)	(40.6)*	(8.1)	(4.2)	(12.1)*
5	1.3	21.6	11.0	50.9	37.9	10.9	70.7
	(4.5)	(5.7)	(6.8)	(30.3)*	(8.2)	(3.9)	(10.7)*
6	6.1	20.3	7.9	97.3	39.8	29.4	27.7
	(4.0)	(8.3)	(7.7)	(126.4)*	(8.7)	(10.8)	(29.7)*
7	2.9	19.3	10.9	35.3	41.1	15.8	59.8
	(4.9)	(7.5)	(5.9)	(44.5)*	(8.8)	(8.9)	(22.1)*
8	2.3	19.4	6.4	66.6	40.1	11.6	71.6
	(3.7)	(6.7)	(3.1)	(15.2)*	(8.1)	(6.2)	(16.8)*
9	-5.2	20.0	12.6	39.5	37.2	6.10	83.5
	(4.9)	(6.2)	(7.3)	(27.3)*	(3.4)	(6.0)	(16.1)*
10	5.8	21.5	6.9	73.9	30.6	17.2	38.7
	(4.6)	(8.3)	(6.0)	(41.6)*	(22.1)	(14.5)	(15.8)*

Negative numbers imply the wrist is in ulnar deviation. Wilcoxon singed-rank tests were applied, and significance is highlighted with *p < 0.05.

**Table 4:** Total number of participants (*n*) adopting radial or ulnar deviations at rest, when each WHO was assessed individually.

WHO	Position of the wrist adopted at rest	
	Ulnar deviation (*n*)	Radial deviation (*n*)
1	2	7
2	3	7
3	0	10
4	2	8
5	4	6
6	0	10
7	4	6
8	1	9
9	7	2
10	0	10

Please note that #1 and #9 only had 9 participants in total.

**Table 5:** Mean grip strength with and without WHO and the %R_Gripstrength_ for each WHO across the ten participants, and wrist radial and ulnar deviation measured at maximum grip strength.

WHO	Maximum grip strength		%R grip strength [%]	Flexion and extension (F/E) movement at maximum grip		Radial and ulnar movement at	
	Without	With WHO	With WHO	Without	With WHO	Without	With WHO
	WHO [kg]	[kg]	[°]	WHO [°]	[°]	WHO [°]	[°]
	Mean	Mean	Mean	Mean	Mean	Mean	Mean
	(S.D.)	(S.D.)	(S.D.)	(S.D)	(S.D)	(S.D)	(S.D)
1	25.3	16.8	33.8	-28.2	0.0	-11.0	8.1
	(4.7)	(6.1)	(10.2)**	(15.1)	(6.2)	(8.9)	(9.6)
2	25.0	21.0	16.1	-32.4	-3.4	-15.4	4.8
	(5.5)	(3.7)	(14.9)**	(10.4)	(7.2)	(7.6)	(5.1)
3	23.7	17.4	26.5	-32.0	-1.0	-11.3	5.9
	(7.8)	(4.8)	(18.0)**	(10.1)	(8.1)	(9.6)	(7.2)
4	25.2	16.6	34.2	-32.2	-0.8	-8.3	7.6
	(5.0)	(6.5)	(10.6)**	(10.3)	(8.8)	(6.0)	(6.8)
5	24.5	19.8	19.3	-31.5	-3.7	-13.7	-2.9
	(5.8)	(5.5)	(15.2)**	(7.7)	(8.7)	(10.6)	(6.7)
6	26.7	26.2	1.7	-26.8	0.7	-8.1	7.0
	(4.4)	(16.8)	(13.4)	(12.4)	(8.4)	(10.1)	(8.2)
7	25.5	20.3	20.6	-33.9	0.2	-12.7	-1.2
	(5.8)	(7.1)	(14.5)**	(7.6)	(8.6)	(12.7)	(9.4)
8	24.5	17.1	30.5	-30.2	-0.2	-12.7	3.5
	(5.0)	(4.2)	(11.6)**	(12.5)	(8.8)	(8.3)	(6.6)
9	25.8	18.4	29.0	-29.4	-0.6	-20.3	-3.6
	(4.3)	(10.5)	(9.7)**	(15.1)	(7.4)	(16.3)	(5.4)
10	26.2	19.9	24.1	-28.9	-6.1	-6.3	3.0
	(5.1)	(4.0)	(12.3)**	(14.9)	(13.3)	(15.9)	(11.4)

*p < 0.05, whilst **p < 0.001. Negative numbers represent wrist ulnar deviation and extension, positive represent radial deviation, and flexion.

**Table 6:** Distribution of each of the participant’s wrist movement in flexion (F), extension (E), ulnar (U), and radial (R) deviations while holding their maximum grip strength with and without each WHO.

WHO	Total Participants (*N*)	Position of the wrist at maximum grip strength						
		Without WHO		With WHO				
		Ulnar (*n*)	Extension (*n*)	Ulnar (*n*)	Radial (*n*)	Neutral coronal (*n*)	Flexion (*n*)	Extension (*n*)
1	9	8	9	2	7	0	5	4
2	10	9	10	2	8	0	3	7
3	10	10	10	2	8	0	4	6
4	10	10	10	2	8	0	4	6
5	10	9	10	7	3	0	5	5
6	10	7	9	3	7	0	7	3
7	10	9	10	5	5	0	4	6
8	10	10	10	3	7	0	4	6
9	9	7	9	6	3	0	4	5
10	10	7	9	1	6	3	2	8

The total participants that adopted each position is indicated by n.

## Data Availability

The raw data supporting the conclusions of this article will be made available by the authors on request, without undue reservation.
